# A Japan nationwide web‐based survey of estimation on patients for renal denervation based on blood pressure level and the number of antihypertensives (J‐NEEDs survey)

**DOI:** 10.1111/jch.14339

**Published:** 2021-08-24

**Authors:** Hideaki Kagitani, Shoko Hayashi, Satsuki Hanamura, Keisuke Ozawa, Daisuke Kobayashi, Shunsuke Hiki, Kazuomi Kario

**Affiliations:** ^1^ Clincal Development Department Terumo Corporation Tokyo Japan; ^2^ Interventional Systems Division, Cardiac and Vascular Company Terumo Corporation Tokyo Japan; ^3^ Division of Cardiovascular Medicine Department of Medicine Jichi Medical University School of Medicine Tochigi Japan

**Keywords:** antihypertensive medication, blood pressure control status, home blood pressure, Japan, National survey

## Abstract

Catheter‐based renal denervation (RDN) is currently being developed as a new complementary treatment option for hypertension. RDN has not yet received approval in Japan and so the number of possible candidates for RDN in Japan also remains unknown. A total of 10 756 hypertensive patients who regularly visit medical institutions and reported their latest home blood pressure (BP) values were identified from registrants at an online research company. They filled out a survey regarding their prescribed antihypertensives and latest BP values in March 2020 in Japan. The mean age of the patients was 61.3 years old (83.5% male). According to JSH 2019, the prevalence of resistant hypertension (RHT) was estimated to be 1.4% (0.52% having an office BP of 140/90 mm Hg or more while taking three antihypertensives, including diuretics; 0.84% taking four or more antihypertensives regardless of BP level). Assuming the indication for RDN was RHT with morning home systolic BP (HSBP) ≥ 135 mm Hg and office systolic BP (OSBP) ≥ 140 mm Hg, the number of candidates for RDN was estimated to be approximately 340 000 and 372 000, respectively. When hypertensive patients prescribed three or more, two, one, and no antihypertensives were included, the estimated number based on uncontrolled HSBP and OSBP cumulatively increased 2.6, 14.2, 40.6, and 58.0‐fold; 1.8, 8.6, 25.3, and 36.4‐fold, respectively. These findings revealed that a substantial number of hypertensive patients are unable to adequately control their BP level with existing treatments, and new complemental therapies, such as RDN, would alleviate the burden of hypertension in this population.

## 1 INTRODUCTION

Management of hypertension is essential to prevent cardiovascular (CV) diseases. The Japanese Society of Hypertension Guidelines for the Management of Hypertension (JSH) in 2014 selected calcium channel blockers (CCBs), angiotensin II receptor blockers (ARBs), angiotensin‐converting enzyme inhibitors (ACEi), and diuretics as the first‐choice drugs for hypertensive patients without compelling indications.^1^ However, according to the latest National Health and Nutrition Survey 2018, there has been no clinically significant improvement in office blood pressure (BP) over the last 10 years.^2^ JSH 2019 was issued 5 years after JSH 2014 and adopted the same first‐choice drugs.^3^ It is necessary to understand to what extent the antihypertensives prescribed actually follow the guidelines.

Another critical point in JSH 2014 was that home BP was prioritized more than office BP.^1^ Multiple clinical studies have reported that home BP has greater predictive power for CV event rates than office BP.^4^, ^5^, ^6^, ^7^, ^8^ However, no research has nationally examined home BP in recent years. Therefore, it is necessary to understand the current status of home BP to determine how many patients potentially need additional treatment.

Catheter‐based renal denervation (RDN) was developed to improve BP mainly among patients diagnosed with resistant hypertension. Early clinical trials, such as SYMPLICITY HTN‐1^9^, ^10^ and SYMPLICITY HTN‐2^11^ showed significant improvements in BP. Unfortunately, SYMPLICITY HTN‐3, a subsequent comparative study with the sham group, failed to show a significant reduction in BP.^12^ Subsequently, ablation techniques and devices have improved, and SPYRAL HTN‐Off MED,^13^, ^14^ and RADIANCE‐HTN SOLO,^15^, ^16^ double‐blind comparative studies with the sham group, showed some effectiveness. Meanwhile, in a comparative study with a sham group and patients with moderate uncontrolled hypertension who were on one to three antihypertensives, early results for SPYRAL HTN‐ON MED^17^ showed a statistically greater improvement of BP at 6 months after RDN. In a RADIENCE‐HTN TRIO^18^ study in which patients with resistant hypertension were randomly assigned to a sham group and an RDN group, the RDN group also showed a significantly larger reduction in BP after 2 months. These results suggest that RDN is a promising new option for the management of both resistant and uncontrolled hypertension, including treatment‐naïve hypertensive patients.

Although some pre‐market clinical trials are on‐going in Japan, the number of candidate hypertensive patients for RDN is unknown. The JAMP study, which enrolled hypertensive patients from 2009 to 2015, simulated the number of patients with resistant hypertension as an indication of RDN.^19^ However, there is no up‐to‐date information on the status of BP control considering actual prescribed antihypertensives and recent clinical evidence about RDN.

This study estimated the number of possible candidate hypertensive cases for RDN in Japan based on the current status of antihypertensive medication use and BP control status.

## 2 METHODS

### 2.1 Study population

An electronic survey was conducted in March 2020 with hypertensive patients registered with the marketing research firm Macromill Carenet to collect information on hypertensive outpatients in Japan (https://www.umin.ac.jp/ctr/index.htm: UMIN000039726). The inclusion criteria were the patients who regularly visit medical institutions for hypertension treatment with or without antihypertensives. The exclusion criteria were under 18 or over 80 years old at the time of the response. The survey collected age, sex, resident area, comorbidities, visit frequency for hypertension treatment, class of prescribed antihypertensives, total number of drugs taken per day, and the most recent home and office BP values. Informed consent was obtained from the patients before their responses. All the information reported by the patients online was anonymized and stored in a database. A total of 10 756 patients who responded with their latest home BP values were used in the analysis. This study was approved for ethical review by the Terumo Corporation (approval number: CR19‐R049).

### 2.2 The number of prescribed antihypertensives

The number of antihypertensives per day was classified as none, one, two, and three or more, based on the class of drugs.

### 2.3 Home and office BP

The patients were asked to report the latest two morning home BP readings measured using their own BP monitoring device before taking antihypertensives, and the mean of morning BP was calculated for analysis. In addition, the patients were asked to report the latest office BP measured based on the method chosen by their physician's discretion. For the 950 patients with unreported office BP (8.8%), we extrapolated the median office BP values calculated by the sex and age of the patients who reported their office BP values. Therefore, both home and office BP were analyzed among all patients.

Resistant hypertension was defined in accordance with JSH 2019 as having an office BP of 140/90 mm Hg or more while taking three or more antihypertensive medications, including diuretics, or as taking four or more antihypertensive medications regardless of BP level.^3^

Home systolic BP was classified as < 125 mm Hg, ≥ 125 to < 135 mm Hg, ≥ 135 to < 145 mm Hg, ≥ 145 to < 155 mm Hg, and ≥ 155 mm Hg, and office systolic BP was classified as < 130 mm Hg, ≥ 130 to < 140 mm Hg, ≥ 140 to < 150 mm Hg, ≥ 150 to < 160 mm Hg, and ≥ 160 mm Hg. BP Phenotype was classified as well‐controlled (office BP is < 140/90 mm Hg and home BP is < 135/85 mm Hg), white coat hypertension (office BP is ≥ 140 mm Hg and/or 90 mm Hg and home BP is < 135 mm Hg/85 mm Hg), masked hypertension (office BP is < 140 mm Hg/90 mm Hg and home BP is ≥ 135 mm Hg and/or 85 mm Hg), and sustained hypertension (office BP is ≥ 140 mm Hg and/or 90 mm Hg and home BP is ≥ 135 mm Hg and/or 85 mm Hg).

### 2.4 Reference studies to compare the currently prescribed drugs and BP level

Because this was a cross‐sectional study, four preceding studies^20^, ^21^, ^22^, ^23^, ^24^ were used to compare previous antihypertensives prescribed and BP management status (Supplementary 1). In addition, three preceding studies^25^, ^26^, ^27^ were used to identify changes in home and office BP based on antihypertensive drug treatment (Supplementary 2).

### 2.5 Estimation of the number of hypertensive patients

JSH 2019 estimated the total number of hypertensive patients in Japan to be 43 million.^3^ It was multiplied by the frequency of each BP level or BP phenotype identified in this study to estimate the number of hypertensive patients in each group.

### 2.6 Statistical analyses

SAS 9.4 (SAS Institute, North Carolina, USA) was used for statistical analysis. Frequencies and percentages were calculated for categorical variables, and means and standard deviations (SD) were calculated for continuous variables. The chi‐square test was used for categorical variables, and the unpaired t‐test and analysis of variance with Bonferroni as ad hoc tests were used for continuous variables to compare between groups. All significance levels were set at 5% (two‐sided).

## 3 RESULTS

Males accounted for 81.2% of the population, the mean age was 62.2 ± 10.0 years, morning home BP was 134.7 ± 13.8/83.1 ± 11.4 mm Hg, and office BP was 135.3 ± 13.5/82.8 ± 10.9 mm Hg. CCBs was the most frequently prescribed antihypertensives (66.0%), followed by ACEi/ARBs (46.0%), vasodilator (8.6%), and diuretics (7.1%) (Table 1).

**TABLE 1:** The characteristics ofhypertensive outpatients

	**Total**	**No antihypertensives**	**At least 1 antihypertensives**	***p***
	*n* = 10 756	*n* = 3002	*n* = 7754	
**Proportion of each group**	100.0%	27.9%	72.1%	
**Male**	81.2%	83.5%	80.3%	<.001
**Age [years] (mean ±sd)**	62.2 ± 10.0	61.3 ± 9.9	62.5 ± 10.0	<.001
**Medical history**				
Hyperlipidemia	45.4%	43.6%	46.0%	.026
Diabetes mellitus	25.0%	27.0%	24.3%	.003
Cardiovascular disease	21.7%	22.2%	21.5%	.442
ASCVD	20.3%	21.0%	20.0%	.272
Coronary artery disease	11.5%	12.2%	11.2%	.138
Stroke	9.5%	10.4%	9.2%	.051
Aortic aneurysm/dissection, PAD	5.1%	5.1%	5.1%	.896
Heart failure	6.3%	6.7%	6.2%	.354
Chronic kidney disease	6.5%	6.4%	6.5%	.874
**Total number of drugs (mean ±sd)**	5.6 ± 6.1	5.8 ± 6.4	5.5 ± 5.9	.004
**Proportion of pts with 6+ drugs**	35.1%	36.1%	34.7%	.186
**Antihypertensive medication**				
Total number of antihypertensives (mean ±sd)	–	–	1.4 ± 0.7	
CCBs	–	–	66.0%	
ACEi/ARB	–	–	46.0%	
Diuretics	–	–	7.1%	
Thiazide diuretics	–	–	4.6%	
MR blocker	–	–	2.0%	
Loop diuretics	–	–	1.1%	
Alpha blocker	–	–	2.9%	
Beta blocker	–	–	6.1%	
Alpha beta blocker	–	–	3.2%	
Direct renin inhibitor	–	–	0.5%	
Vasodilator	–	–	8.6%	
Central alpha‐2 adrenergic agonist	–	–	0.5%	
**Morning home blood pressure**				
Systolic BP [mm Hg] (mean ±sd)	134.7 ± 13.8	135.7 ± 14.2	134.3 ± 13.6	<.001
Diastolic BP [mm Hg] (mean ±sd)	83.1 ± 11.4	84.0 ± 11.9	82.7 ± 11.2	<.001
Uncontrolled BP (*≥*125 or *≥*75 mm Hg)	91.2%	91.7%	90.9%	.206
Uncontrolled BP (*≥*135 or *≥*85 mm Hg)	58.6%	62.9%	56.9%	<.001
**Office blood pressure**				
Systolic BP [mm Hg] (mean ±sd)	135.3 ± 13.5	135.9 ± 14.0	135.0 ± 13.3	.001
Diastolic BP [mm Hg] (mean ±sd)	82.8 ± 10.9	83.7 ± 11.4	82.5 ± 10.6	<.001
Uncontrolled BP (*≥*130 or *≥*80 mm Hg)	82.4%	84.0%	81.8%	<.001
Uncontrolled BP (*≥*140 or *≥*90 mm Hg)	38.5%	42.6%	36.9%	<.001

*Abbreviations*: ACEi, Angiotensin‐converting enzyme inhibitors; ARB, Angiotensin II Receptor Blocker; ASCVD, Atherosclerotic Cardiovascular Disease; BP, Blood pressure; CCBs, calcium channel blockers; PAD, Peripheral Artery Disease; SD, Standard deviation.

The total number of medications including antihypertensives per day was 6.2 ± 6.1 for the two antihypertensives group and 9.0 ± 6.9 for the three or more antihypertensives group, which were significantly higher compared with the one antihypertensive group (4.8 ± 5.6 drugs) (*p* < .05, *p* < .01, respectively). CCBs and ACEi/ARB were the first and second most commonly prescribed in either group. Diuretics were prescribed to 47.5% of patients in the three or more antihypertensives group, which is about five times greater than in the two antihypertensives group. Resistant hypertension based on JSH 2019 accounted for 1.4% of all hypertensive patients. The prevalence increased to 1.8% when a newly recommended control target of < 130/80 mm Hg is adopted according to 2017 American College of Cardiology/AHA guideline.^28^ All comorbidities were more common in the resistant hypertension group than the others. In particular, diuretics were prescribed in 84.9% of the patients, Thiazide diuretic in 52.1%, MR blocker in 35.6%, and loop diuretic in 24.0% (Table 2).

**TABLE 2:** The characteristics of hypertensive outpatients based on the number of antihypertensives

	**1 antihypertensives**	**2 antihypertensives**	**3+ antihypertensives**	***p***	**Resistant hypertension** ^a^
	*n* = 5114	*n* = 2177	*n* = 463		n = 146
**Proportion of each group** ^b^	47.5%	20.2%	4.3%		1.4%
**Male**	78.0%	84.2%	87.7%	<.001	83.6%
**Age [years] (mean ±sd)**	62.6 ± 9.9	62.4 ± 10.0	61.8 ± 11.4	.217	58.0 ± 13.8
**Medical history**					
Hyperlipidemia	45.9%	46.3%	46.0%	.950	53.4%
Diabetes mellitus	23.5%	24.9%	30.2%	.004	39.7%
Cardiovascular disease	19.9%	21.9%	38.7%	<.001	42.5%
ASCVD	18.6%	20.2%	35.0%	<.001	38.4%
Coronary artery disease	10.0%	11.9%	21.2%	<.001	26.7%
Stroke	8.9%	8.4%	16.4%	<.001	25.3%
Aortic aneurysm/dissection, PAD	4.3%	5.3%	12.7%	<.001	18.5%
Heart failure	5.2%	6.7%	15.1%	<.001	26.0%
Chronic kidney disease	5.4%	7.9%	13.0%	<.001	21.2%
**Total number of drugs (mean ±sd)**	4.8 ± 5.6^**^,^***^	6.2 ± 6.1^*^,^***^	9.0 ± 6.9^*^,^**^	<.001	11.2 ± 8.7
**Proportion of pts with 6+ drugs**	29.6%	40.6%	63.5%	<.001	70.6%
**Antihypertensive medication**					
CCBs	55.0%	86.7%	90.7%	<.001	88.4%
ACEi/ARB	30.1%	76.0%	80.4%	<.001	76.0%
Diuretics	2.4%	9.7%	47.5%	<.001	84.9%
Thiazide diuretics	1.5%	6.5%	30.7%	<.001	52.1%
MR blocker	0.7%	1.9%	16.6%	<.001	35.6%
Loop diuretics	0.3%	1.5%	8.9%	<.001	24.0%
Alpha blocker	0.5%	4.0%	23.5%	<.001	30.8%
Beta blocker	1.8%	9.6%	36.3%	<.001	40.4%
Alpha beta blocker	0.8%	4.9%	21.0%	<.001	19.2%
Direct renin inhibitor	0.2%	0.4%	3.5%	<.001	6.9%
Vasodilator	8.8%	7.4%	13.4%	<.001	15.1%
Central alpha‐2 adrenergic agonist	0.2%	0.5%	3.0%	<.001	6.9%
**Morning home blood pressure**					
Systolic BP [mm Hg] (mean ±sd)	134.1 ± 12.0^***^	134.6 ± 14.3	136.0 ± 16.4^*^	.008	140.5 ± 20.0
Diastolic BP [mm Hg] (mean ±sd)	82.8 ± 11.1	82.5 ± 11.0	82.0 ± 12.9	.255	82.9 ± 14.2
Uncontrolled HBP (*≥*125 or *≥*75 mm Hg)	91.5%	90.0%	89.0%	.045	92.5%
Uncontrolled HBP (*≥*135 or *≥*85 mm Hg)	56.9%	56.5%	58.8%	.675	67.8%
**Office blood pressure**					
Systolic BP [mm Hg] (mean ±sd)	135.0 ± 12.8^***^	134.6 ± 13.3^***^	136.9 ± 18.0^*,**^	.004	145.6 ± 20.4
Diastolic BP [mm Hg] (mean ±sd)	82.7 ± 10.4	82.1 ± 10.5	81.8 ± 12.9	.027	84.8 ± 14.3
Uncontrolled OBP (*≥*130 or *≥*80 mm Hg)	82.6%	80.8%	78.0%	.001	87.7%
Uncontrolled OBP (*≥*140 or *≥*90 mm Hg)	37.1%	35.5%	41.7%	.038	69.9%

*Abbreviations*: ACEi, Angiotensin‐converting enzyme inhibitors; ARB, Angiotensin II Receptor Blocker; ASCVD, Atherosclerotic Cardiovascular Disease; BP, Blood pressure; CCBs, calcium channel blockers; PAD, Peripheral Artery Disease; SD, Standard deviation.

^a^ Resistant hypertension is defined as having an office blood pressure of 140/90 mm Hg or more while taking three antihypertensive medications, including diuretics, or taking four or more antihypertensive medications regardless of blood pressure level.

^b^ The denominator of the proportion is total patients of this survey (*n* = 10756).

* Significantly different compared with one antihypertensives (*p* < .05).

** Significantly different compared with two antihypertensives (*p* < .05).

*** Significantly different compared with three or more antihypertensives (*p* < .05).

Table 3 shows the achievement status on the target levels of BP by age, as presented in JSH 2019. In adults younger than 75 years of age, about 90% of patients did not achieve the target level of home BP control (< 125/75 mm Hg), regardless of the number of antihypertensives. It was also the same in resistant hypertension. In patients aged 75 and over, more than half did not achieve the target level of home BP control (< 135/85 mm Hg). Approximately 80% of adults younger than 75 years old did not meet their target level in terms of office BP (< 130/80 mm Hg), with significant differences by the number of antihypertensives (*p* < .05). In contrast, about one‐third of patients aged 75 and over did not meet the target level of office BP (< 140/90 mm Hg).

**TABLE 3:** ’t achieve target level of blood pressure in Japan (age stratification)

	**Aged below 75**				**Aged 75 or higher**			
**Morning home BP (mm Hg)**	**SBP** *≥* **125 or DBP** *≥* **75**			***p***	**SBP** *≥* **135 or DBP** *≥* **85**			***p***
	***N***	***n***	**%**		***N***	***n***	**%**	
**Resistant hypertension** ^a^	138	127	92.0%	–	8	6	75.0%	–
**3+ antihypertensives**	422	376	89.1%	.071	41	25	61.0%	.715
**2 antihypertensives**	1972	1782	90.4%		205	108	52.7%	
**1 antihypertensives**	4603	4225	91.8%		511	275	53.8%	
**No antihypertensives**	2753	2528	91.8%		249	140	56.2%	
**Total**	9750	8911	91.4%	–	1006	548	54.5%	–

	**Aged below 75**				**Aged 75 or higher**			
**Office BP (mm Hg)**	**SBP** *≥* **130 or DBP** *≥* **80**			***p***	**SBP** *≥* **140 or DBP** *≥* **90**			***p***
	**N**	**n**	**%**		**N**	**n**	**%**	
**Resistant hypertension** ^a^	138	122	88.4%	–	8	5	62.5%	–
**3+ antihypertensives**	422	333	78.9%	.002	41	16	39.0%	.262
**2 antihypertensives**	1972	1595	80.9%		205	65	31.7%	
**1 antihypertensives**	4603	3810	82.8%		511	170	33.3%	
**No antihypertensives**	2753	2324	84.4%		249	98	39.4%	
**Total**	9750	8062	82.7%	–	1,006	349	34.7%	–

*Abbreviations*: BP, Blood pressure; DBP, Diastolic blood pressure; SBP, Systolic blood pressure.

^a^ Resistant hypertension is defined as having an office blood pressure of 140/90 mm Hg or more while taking three antihypertensive medications, including diuretics, or taking four or more antihypertensive medications regardless of blood pressure level.

The BP control status in resistant hypertension and by the number of antihypertensives is shown in Table 4. The percentages of uncontrolled morning home systolic BP equal to or higher than 135 mm Hg were 58.2% in resistant hypertension, and 47.5%, 45.4%, 43.8%, and 49.5% for the three or more, two, one, and no antihypertensives groups, respectively. The proportion of office systolic BP equal to or higher than 140 mm Hg were 63.7% in resistant hypertension, and 36.9%, 29.0%, 30.2%, and 34.3% for the three or more, two, one, and no antihypertensives groups, respectively. Masked hypertension according to the BP phenotype was 13.0% for resistant hypertension, and 25.7%, 28.5%, 27.1%, and 25.8% for the three or more, two, one, and no antihypertensives groups, respectively. Considering the combination of home and office systolic BP, the 47.9% of resistant hypertension showed home systolic BP ≥ 135 mm Hg and office systolic BP ≥ 140 mm Hg (Supplementary 3).

**TABLE 4:** Distribution of blood pressure level among hypertensive outpatients in Japan

**Morning home SBP [mm Hg]**	**All (N)**	**<125**	*≥* **125 to < 135**	*≥* **135 to < 145**	*≥* **145 to < 155**	*≥* **155**	***p***	**Uncontrolled HSBP** ^c^ [HSBP ≥135 mm Hg]
**Resistant hypertension** ^a^	146	15.8%	26.0%	25.3%	14.4%	18.5%	–	58.2%
**3+ antihypertensives**	463	21.4%	31.1%	21.4%	15.6%	10.6%	<.001	47.5%
**2 antihypertensives**	2,177	21.8%	32.8%	26.0%	11.6%	7.7%		45.4%
**1 antihypertensives**	5,114	20.9%	35.3%	26.5%	10.7%	6.6%		43.8%
**No antihypertensives**	3,002	18.3%	32.2%	27.2%	13.7%	8.6%		49.5%
**Total**	10,756	20.4%	33.7%	26.4%	11.9%	7.5%	–	45.9%

**Office SBP [mm Hg]**	**All (N)**	**<130**	**≥130 to < 140**	**≥ 140 to < 150**	**≥ 150 to < 160**	**≥ 160**	***p***	**Uncontrolled OSBP** ^d^ [OSBP ≥140 mm Hg]
**Resistant hypertension** ^a^	146	16.4%	19.9%	26.7%	13.7%	23.3%	–	63.7%
**3+ antihypertensives**	463	31.1%	32.0%	16.0%	8.4%	12.5%	<.001	36.9%
**2 antihypertensives**	2,177	32.0%	39.0%	17.3%	6.6%	5.1%		29.0%
**1 antihypertensives**	5,114	29.1%	40.7%	17.4%	8.1%	4.8%		30.2%
**No antihypertensives**	3,002	27.1%	38.6%	18.5%	9.4%	6.4%		34.3%
**Total**	10,756	29.2%	39.4%	17.6%	8.2%	5.6%	–	31.4%

**BP phenotype** ^b^	**All (N)**	**Well‐controlled** [OBP < 140/90 mm Hg and HBP < 135/85 mm Hg]	**White coat Hypertension** [OBP ≥140 and/or 90 mm Hg and HBP < 135/85 mm Hg]	**Masked Hypertension** [OBP < 140/90 mm Hg and HBP ≥135 and/or 85 mm Hg]	**Sustained Hypertension** [OBP ≥140 and/or 90 mm Hg and HBP ≥135 and/or 85 mm Hg]	***p***	**Masked + Sustained hypertension**
**Resistant hypertension** ^a^	146	17.1%	15.1%	13.0%	54.8%	–	67.8%
**3+ antihypertensives**	463	32.6%	8.6%	25.7%	33.0%	<.001	58.7%
**2 antihypertensives**	2,177	36.0%	7.5%	28.5%	28.0%		56.5%
**1 antihypertensives**	5,114	35.8%	7.3%	27.1%	29.7%		56.9%
**No antihypertensives**	3,002	31.5%	5.5%	25.8%	37.1%		62.9%
**Total**	10,756	34.5%	6.9%	27.0%	31.6%	–	58.6%

*Abbreviations*: BP, Blood pressure; HBP; Home blood pressure; HSBP, Home systolic blood pressure; OBP, Office blood pressure; OSBP, Office systolic blood pressure; SBP, Systolic blood pressure.

^a^ Resistant hypertension is defined as having an office blood pressure of 140/90 mm Hg or more while taking three antihypertensive medications, including diuretics, or taking four or more antihypertensive medications regardless of blood pressure level.

^b^ BP Phenotype is classified as well‐controlled (office blood pressure is < 140/90 mm Hg and home blood pressure is < 135/85 mm Hg), white coat hypertension (office blood pressure is ≥140 mm Hg and/or 90 mm Hg and home blood pressure is < 135 mm Hg/85 mm Hg), masked hypertension (office blood pressure is < 140 mm Hg/90 mm Hg and home blood pressure is ≥135 mm Hg and/or 85 mm Hg), and sustained hypertension (office blood pressure is ≥140 mm Hg and/or 90 mm Hg and home blood pressure is ≥135 mm Hg and/or 85 mm Hg).

^c^ Uncontrolled HSBP is defined based on only home systolic blood pressure of 135 mm Hg or more.

^d^ Uncontrolled OSBP is defined based on only office systolic blood pressure of 140 mm Hg or more.

Table 5 shows the estimated number of hypertensive patients based on systolic BP level or BP phenotype, assuming that the total number of hypertensive patients in Japan is 43 million. The number of patients with morning home systolic BP equal to or higher than 135 mm Hg was approximately 340 000 in resistant hypertension. When hypertensive patients prescribed three or more, two, one, and no antihypertensives were included, the cumulative number of patients with the same home systolic BP criterion increased to 881 000, 4 824 000, 13 778 000, and 19 719 000, respectively. Similarly, the number of patients with office systolic BP equal to or higher than 140 mm Hg was 372 000 in resistant hypertension. When hypertensive patients prescribed three or more, two, one, and no antihypertensives were included, the cumulative number of patients with the same office BP criterion increased to 683 000, 3 207 000, 9 402 000, and 13 518 000, respectively.

**TABLE 5:** Estimated number of hypertensive patients per blood pressure level in Japan

**Home SBP [mm Hg]**	**All**	**< 125**	**≥ 125 to < 135**	**≥ 135 to < 145**	**≥ 145 to < 155**	**≥ 155**	**Uncontrolled HSBP** ^c^	
							[HSBP ≥ 135 mm Hg]	
							**Subtotal**	**Cumulative**
**Resistant hypertension** ^a^	583 674	91 949	151 915	147 917	83 953	107 940	339 810	339 810
**3+ antihypertensives**	1 850 967	395 779	575 679	395 779	287 839	195 891	879 509	881 060
**2 antihypertensives**	8 703 142	1 898 940	2 854 407	2 266 735	1 011 435	671 625	3 949 795	4 823 584
**1 antihypertensives**	20 444 589	4 269 617	7 211 975	5 424 972	2 194 775	1 343 250	8 962 997	13 778 314
**No antihypertensives**	12 001 302	2 194 775	3 869 840	3 270 175	1 639 085	1 027 427	5 936 687	19 718 958
**Total**	43 000 000	8 759 111	14 511 900	11 357 661	5 133 135	3 238 193	19 718 958	‐

**Office SBP [mm Hg]**	**All**	**< 130**	**≥130 to < 140**	**≥ 140 to < 150**	**≥ 150 to < 160**	**≥ 160**	**Uncontrolled OSBP** ^d^	
							[OSBP ≥ 140 mm Hg]	
							**Subtotal**	**Cumulative**
**Resistant hypertension** ^a^	583 674	95 946	115 935	155 913	79 955	135 924	371 792	371 792
**3+ antihypertensives**	1 850 967	575 679	591 670	295 835	155 913	231 871	683 618	683 007
**2 antihypertensives**	8 703 142	2 786 445	3 394 106	1 507 159	575 679	439 755	2 522 592	3 206 918
**1 antihypertensives**	20 444 589	5 956 675	8 315 359	3 550 019	1 651 078	971 458	6 172 555	9 401 629
**No antihypertensives**	12 001 302	3 258 181	4 629 416	2 222 759	1 127 371	763 574	4 113 704	13 518 075
**Total**	43 000 000	12 576 980	16 930 550	7 575 772	3 510 041	2 406 657	13 518 075	–

**BP phenotype** ^b^	**All**	**Well‐controlled**	**White coat Hypertension**	**Masked Hypertension**	**Sustained Hypertension**		**Masked + Sustained Hypertension**	
		[OBP < 140/90 mm Hg and HBP < 135/85 mm Hg]	[OBP ≥ 140 and/or 90 mm Hg and HBP < 135/85 mm Hg]	[OBP < 140/90 mm Hg and HBP ≥ 135 and/or 85 mm Hg]	[OBP ≥ 140 and/or 90 mm Hg and HBP ≥ 135 and/or 85 mm Hg]		**Subtotal**	**Cumulative**
**Resistant hypertension** ^a^	583 674	99 944	87 951	75 958	319 821		395 779	395 779
**3+ antihypertensives**	1 850 967	603 663	159 911	475 734	611 659		1 087 393	1 086 518
**2 antihypertensives**	8 703 142	3 134 251	651 636	2 482 614	2 434 641		4 917 255	6 003 793
**1 antihypertensives**	20 444 589	7 323 912	1 495 165	5 544 905	6 080 606		11 625 511	17 636 764
**No antihypertensives**	12 001 302	3 785 887	663 630	3 098 271	4 453 514		7 551 785	25 185 583
**Total**	43 000 000	14 847 713	2 970 342	11 601 525	13 580 420		25 185 583	–

*Abbreviations*: BP, Blood pressure; HBP; Home blood pressure; HSBP, Home systolic blood pressure; OBP, Office blood pressure; OSBP, Office systolic blood pressure; SBP, Systolic blood pressure.

^a^ Resistant hypertension is defined as having an office blood pressure of 140/90 mm Hg or more while taking three antihypertensive medications, including diuretics, or taking four or more antihypertensive medications regardless of blood pressure level.

^b^ BP Phenotype is classified as well‐controlled (office blood pressure is < 140/90 mm Hg and home blood pressure is < 135/85 mm Hg), white coat hypertension (office blood pressure is ≥140 mm Hg and/or 90 mm Hg and home blood pressure is < 135 mm Hg/85 mm Hg), masked hypertension (office blood pressure is < 140 mm Hg/90 mm Hg and home blood pressure is ≥135 mm Hg and/or 85 mm Hg), and sustained hypertension (office blood pressure is ≥140 mm Hg and/or 90 mm Hg and home blood pressure is ≥135 mm Hg and/or 85 mm Hg).

^c^ Uncontrolled HSBP is defined based on only home systolic blood pressure of 135 mm Hg or more.

^d^ Uncontrolled OSBP is defined based on only office systolic blood pressure of 140 mm Hg or more.

The number of possible candidate patients for RDN according to the inclusion criteria of Spyral HTN‐OFF and HTN‐ON MED was estimated on the basis of office BP, home BP, and the number of antihypertensives. The candidates had to have a systolic BP between 150 and 180 mm Hg, diastolic BP of 90 mm Hg or higher, and 24‐h ambulatory systolic BP between 140 and 170 mm Hg. Because 24‐h ambulatory BP was not measured in this study, it was replaced with a home systolic BP of between 140 and 170 mm Hg. Consequently, 7.0% of hypertensive patients prescribed no antihypertensives and 4.9% of hypertensive patients prescribed at least one antihypertensive, which is 3 010 000 and 2 107 000 hypertensive patients, respectively, were candidates for RDN.

## 4 DISCUSSION

This study is the latest nationwide survey to determine not only the medication status including antihypertensives but also home and office BP control in Japanese hypertensive patients. The majority of younger hypertensive patients did not meet the JSH 2019 target levels of home BP control (< 125/75 mm Hg). Masked hypertension was found in one‐quarter of hypertensive patients. The most commonly prescribed antihypertensive was CCBs, followed by ACEi/ARBs. Patients taking two or more antihypertensives took an average of six or more drugs per day. Given the total number of hypertensive patients in Japan, the estimated number of candidates for RDN with resistant hypertension based on uncontrolled home systolic BP and office systolic BP was 340 000 and 372 000, respectively. It increased 58.0‐fold and 36.4‐fold when including all uncontrolled hypertensive patients, including treatment‐naïve hypertensive patients.

### 4.1 Current status of home BP and its changes over 20 years

In this study, mean home and office BP were 134.3/82.7 mm Hg and 135.0/82.5 mm Hg among hypertensive patients with at least one antihypertensives, respectively. Approximately 60% of them showed uncontrolled morning home (≥ 135 or 85 mm Hg) and 40% showed uncontrolled office (≥ 140 or 90 mm Hg) BP. These results were consistent regardless of the number of prescribed antihypertensives. Four previous large observational studies, J‐HOME,^20^, ^21^, ^22^ J‐MORE,^23^ and J‐HOP,^7^, ^8^ and the study using claim data^24^ were used to examine changes in BP control status and antihypertensives prescribed and over time (See Supplementary 1). In previous studies that investigated both home and office BP, the mean home and office BP were 136.8/79.3 mm Hg and 142.8/80.6 mm Hg in J‐HOME study,^20^, ^21^, ^22^ 139.8/81.7 mm Hg and 143.0/80.7 mm Hg in J‐MORE study,^23^ and 138.4/79.1 mm Hg and 141.3/81.2 mm Hg in J‐HOP study.^7^, ^8^ Although there was no major difference in patients’ characteristics between the current and previous studies, with more emphasis placed on home BP in JSH 2014, the improvement in home BP was small. In particular, approximately 90% of hypertensive patients aged < 75 years did not achieve the target level of home BP (< 125/75 mm Hg) set by JSH2019, regardless of the number of antihypertensives. One potential factor for the current situation, clinical inertia, where intensification of treatment is needed but not provided, has been reported.^29^ It also has been reported that more antihypertensive prescriptions are accompanied by challenges such as poor adherence,^30^ side effects,^31^ and prescribing cascade that responds to the side effects of more medicine with further increases.^32^ A medicine increase is known to cause problems with polypharmacy, especially when six or more drugs are prescribed, which can lead to increased side effects.^33^ In this study, it should be noted that the total number of medications per day in patients prescribed two or more antihypertensives exceeded six drugs. Therefore, in patients taking more than two antihypertensives, increase in antihypertensives may not be always appropriate. Also, since younger adults are suggested to be one of /responders to RDN from pathophysiological view point^34^ and would benefit to reduce CV disease risk for longer period if long‐term efficacy is established, they are expected to be main population indicated for RDN.

### 4.2 Masked hypertension and its treatment

In this study, masked hypertension (defined as morning home BP ≥ 135 or 85 mm Hg and normal office BP < 140 and 90 mm Hg, regardless of the number of antihypertensives, based on JSH 2019) was found in about one‐fourth of all patients and accounted for about 40% of patients with well‐controlled office BP. The prevalence of masked hypertension was higher in this study than in any previous study,^7^, ^8^, ^20^, ^21^, ^22^, ^23^ while the prevalence of sustained hypertension was the lowest. As seen in previous studies^25^, ^26^, ^27^ that assessed the efficacy of antihypertensives on both home and office BP, this can be described as a state of masked uncontrolled hypertension, in which a patient with sustained hypertension is given antihypertensive medication. Although their office BP is lowered, the effect of the antihypertensives does not last until the next morning, so the home BP does not drop completely, and prevalence of masked hypertension eventually increases. These results indicated that diagnosis and treatment of hypertension based on office BP alone may miss the elevated CV disease risk in many hypertensive patients because masked hypertension has been reported to have a higher CV disease risk, along with sustained hypertension.^8^ Therefore, a thorough assessment of home BP is essential, as specified in the JSH guidelines. Because recent studies regarding RDN compared to the sham procedure^13^, ^14^, ^15^, ^16^, ^17^, ^18^, ^35^ reported that RDN lowered BP all day long, its clinical value in masked hypertension is likely to be promising.^34^

### 4.3 Changes in medication therapy based on the JSH guidelines

In this study, CCBs were most commonly prescribed, followed by ACEi/ARBs, confirming the continuation of trends in accordance with JSH guidelines. Diuretics were prescribed for only 7.1% of hypertensive patients taking at least one antihypertensives, despite diuretics being one of the first‐line drugs. They were more frequently prescribed for resistant hypertension (84.9%) and patients with three or more antihypertensives (47.5%) compared with patients with two (9.7%) and one antihypertensives (2.4%). Although the importance of diuretics has been emphasized in updates of the Japanese Society of Hypertension Guidelines for the Management of Hypertension for the last 20 years, diuretic prescription rate in overall patients remained less than 10% both in J‐HOME^20^ that conducted in 2003 and in the current study. Among hypertensive patients with three or more antihypertensives, the diuretic prescription rate was 1.5 times higher in the current study (47.5%) compared to J‐HOME^20^ (31.6%). Nonetheless, hypertension control did not improve at the same rate. In addition, regardless of the fact that the MR blocker is recommended for resistant hypertension by JSH2019, this study revealed that it was prescribed for only one‐third of cases of resistant hypertension. These results indicated that there is still room for improvement in adopting JSH2019.

### 4.4 Estimation of uncontrolled and resistant hypertensive candidate patients for RDN

This study revealed that, depending on the use of home or office BP to define uncontrolled hypertension, the number of candidates for RDN with resistant hypertension was estimated to be at least 340 000 or 372 000, respectively. The estimate increased 40.6‐fold and 25.3‐fold when including uncontrolled hypertensive patients prescribed at least one antihypertensives. Further, when all uncontrolled hypertensive patients included, the estimate soared to 58.0‐fold and 36.4‐fold. As hypertensive patients would need to be carefully screened for RDN by assessing BP control level using 24‐h ambulatory BP monitoring to detect daytime and nocturnal hypertension, confirming adherence to the prescribed antihypertensives, excluding secondary hypertension, and determining the appropriateness of renal artery anatomy, a multidisciplinary approach is necessary for the appropriate application of RDN.^36^ Although the actual number of hypertensive patients who eventually undergo RDN would be much less than the estimate, further consideration will be crucial in the adoption of RDN as a standard of care.

5

This study is highly representative of the actual situation in Japan due to a large number of cases and the fact that information was collected from all over Japan. In addition, because the most recent BP reading measured at home was submitted by the participants, it was considered highly reliable. On the other hand, there are certain limitations. The results cannot be directly applied to countries other than Japan. Since it was a self‐reported internet survey, source verification was not performed and there may have been fewer responses from hypertensive patients who are unfamiliar with the internet and older adults. Another limitation was the lack of an adherence assessment and 24‐h ambulatory BP monitoring which are current standard procedures for confirming eligibility for RDN in clinical studies. Moreover, the number of candidates for RDN was a crude estimate because the prevalence of hypertension by age and sex in the Japanese population was not available. Also, the number of candidates may have been overestimated by at least 12.2% because secondary hypertension^37^, ^38^ was not distinguished or excluded.

## 6 CONCLUSIONS

In conclusions, when the indications for RDN were limited to resistant hypertension with uncontrolled home and office BP, it was estimated that the number of candidates for RDN would be at least 340 000 and 372 000, respectively. The number of candidates might increase more than twenty‐five‐fold when RDN is used to treat patients with at least one antihypertensive and more than thirty‐six‐fold when RDN is used to treat all uncontrolled hypertensive patients. Side effects induced by the dose escalation of antihypertensives was a concern because one‐third of hypertensive patients have already been prescribed six or more medications in total. Therefore, complementary treatment options, such as RDN, are needed for substantial hypertensive patients to improve hypertension control. More research is necessary to comprehensively quantify hypertensive patients who require complementary treatment.

## FUNDING

This study was financially supported by Terumo Corporation.

## CONFLICT OF INTEREST

K. Kario is contracted as the external medical adviser of Terumo Corporation. K. Kario received honoraria from Jimro and Medtronic Japan and research grant from Otsuka Holdings and Otsuka Pharmaceuticals outside the submitted work. H. Kagitani, S. Hayashi, S. Hanamura, K. Ozawa, D. Kobayashi, and S. Hiki are full‐time employees of Terumo Corporation.

## AUTHOUR CONTRIBUTION

Hideaki Kagitani, Shoko Hayashi, Satsuki Hanamura, Keisuke Ozawa, Daisuke Kobayashi, Shunsuke Hiki, and Kazuomi Kario were involved in study design and data interpretation. Hideaki Kagitani was responsible for the data analysis. All authors critically revised the report, commented on drafts of the manuscript, and approved the final report.

## Supporting information

Supplementary information
