# Supplementary material for: A Japan nationwide web‐based survey of estimation on patients for renal denervation based on blood pressure level and the number of antihypertensives (J‐NEEDs survey)
Source: J Clin Hypertens (Greenwich). 2021 Aug 24;23(9):1684–94. doi: 10.1111/jch.14339 (PMC8678654; doi:10.1111/jch.14339)
Supplement: Supplementary file 1 — Supplementary information [file JCH-23-1684-s001.docx]

**Supplementary 1. Previous large studies that investigated both home and office blood pressure and prescribed antihypertensives**

| **Study name** | | **J-HOME^20-22^** | | **J-MORE^23^** | **J-HOP^7,8^** | **Study by Tsuchihashi^24^** | **J-NEEDs survey*** | |
| --- | --- | --- | --- | --- | --- | --- | --- | --- |
| **Type of study** | | Observational study | | Observational study | Observational study | Claim data | Self-reported observational study | |
| **Year for enrollment** | | 2003 - 2004 | | 2004 | 2005-2012 | 2014 - 2015 | 2020 | |
| **Subject settings** | | Primary care settings | | Clinics and hospitals | University hospital | Acute hospitals | Clinics and hospitals | |
| **n** | | 3,303 | | 1,027 | 4,261 | 59,867 | 10,756 | |
| **Inclusion criteria** | | Hypertensive patients with antihypertensives treatment in primary care setting | | Hypertensive outpatients with stable treatment for at least 3 months | Japanese individuals with any of the cardiovascular risk factors: diabetes, dyslipidemia, current smoking (or current chronic obstructive pulmonary disease), chronic kidney disease, atrial fibrillation, metabolic syndrome, and sleep apnea syndrome. | Patients who have outpatient claim for antihypertensives between April 2014 and March 2015. | Hypertensive patients who visit a clinic or hospital at least once per three months | |
| **Exclusion criteria** | |  | |  | Recent history of cardiovascular disease events (within 6 months), current hemodialysis treatment, chronic inflammatory disease, or malignancy |  |  | |
| **Age** | | 66.2±10.5 | | 66.5±10.2 | 64.9±10.9 | 70.0±11.9 | 62.5±10.0 | |
| **Male** | | 44.8% | | 42.0% | 46.9% | 56.9% | 80.3% | |
| **Medical History** | **Cerebrovascular disease** | 16.7% | | 7.4% | - | 19.4% | 9.2% | |
|  | **Ischemic heart disease** | 8.3% | | 13.9% | 12.7% | 52.0% | 11.2% | |
|  | **Diabetes mellitus** | 13.8% | | 15.9% | 24.3% | 24.3% | 24.3% | |
|  | **Renal disease** | unspecified | | 5.0% | - | 10.0% | 6.5% | |
|  | **Dyslipidemia** | 40.5% | | 40.9% | 23.6% | 56.8% | 46.0% | |
| **Proportion of patients with at least one antihypertensives** | | 100.0% | | 94.4% | 79.2% | 100.0% | 72.1% | |
| **Number of antihypertensives** | | 1 drug 48.7%  2 drugs 35.4%  3 drugs 12.3%  4 or more drugs 3.6% | | - | - | 1 drug 41.4%  2 drugs 34.2%  3 drugs 16.5%  4 drugs 6.1%  5 or more drugs 1.7% | 0 drug 27.9%  1 drug 47.5%  2 drugs 20.2%  3 or more drugs 4.3% | |
| **Class of antihypertensives** | **CCBs** | 69.6% | | 71.2% | 50.9% | 66% | 66.0% | |
|  | **ACEi** | 16.7% | | 27.3% | 6.6% | 9.5% | 46.0% | |
|  | **ARB** | 43.6% | | 31.6% | 51.7% | 60% |  |  |
|  | **Diuretics** | All | 3 or more | 12.6% | 26.0% | Unspecified | All | 3 or more |
|  |  | 9.3% | 31.6% |  |  |  | 7.1% | 47.5% |
|  | **Thiazide diuretics** | - | 22.0% | - | - | 10% | 4.6% | 30.7% |
|  | **MR blockers** | - | 5.1% | - | - | 5% | 2.0% | 16.6% |
|  | **Loop diuretics** | - | 7.2% | - | - | 10.5% | 1.1% | 8.9% |
|  | **Alpha blocker** | 13.4% | | 10.6% | 5.0% | 4% | 2.9% | |
|  | **Beta blocker** | 11.7% | | 21.7% | 13.7% | 25% | 6.1% | |
| **Blood pressure**  **(mean±SD mmHg)** | **Morning home SBP** | 136.8 ± 12.5 | | 139.8 ± 14.6 | 138.4±15.8 | N/A | 134.3±13.6 | |
|  | **Morning home DBP** | 79.3 ± 8.8 | | 81.7±10.0 | 79.1±10.0 |  | 82.7±11.2 | |
|  | **Office SBP** | 142.8 ± 14.4 | | 143.0±15.6 | 141.3±16.4 |  | 135.0±13.3 | |
|  | **Office DBP** | 80.6 ± 9.4 | | 80.7±10.1 | 81.2±10.6 |  | 82.5±10.6 | |
| **Proportion of uncontrolled BP** | **Morning home BP**  **(>135 or >85 mmHg)** | 57.6% | | - | 58.0% | N/A | 56.9% | |
|  | **Office BP**  **(>140 or >90 mmHg)** | 48.0% | | 56.4%  (Only SBP) | 53.4% |  | 36.9% | |
| **BP phenotype**** | **Well-controlled** | 23.0% | | - | 27.6% | N/A | 35.7% | |
|  | **White-coat hypertension** | 19.4% | | - | 14.4% |  | 7.4% | |
|  | **Masked hypertension** | 19.0% | | 22.5% | 19.0% |  | 27.4% | |
|  | **Sustained hypertension** | 38.7% | | - | 39.0% |  | 29.4% | |

*CCBs: calcium channel blockers; ACEi: angiotensin converting enzyme inhibitors; ARB: Angiotensin II receptor blockers; SBP: Systolic blood pressure; HT: Hypertension; BP: Blood pressure.*

**The results of J-NEEDs survey are based on the patients with at least one antihypertensives.*

**** *BP Phenotype is classified as well-controlled (office blood pressure is <140/90 mmHg and home blood pressure is <135/85 mmHg), white coat hypertension (office blood pressure is ≥140mmHg and/or 90 mmHg and home blood pressure is <135 mmHg/85 mmHg) ), masked hypertension (office blood pressure is <140 mmHg/90mmHg and home blood pressure is ≥135mmHg and/or 85 mmHg), and sustained hypertension (office blood pressure is ≥140mmHg and/or 90 mmHg and home blood pressure is ≥135mmHg and/or 85 mmHg).*

**Supplementary 2. Previous interventional studies that investigated the changes in both home and office blood pressure by antihypertensives**

| **Study name** | | **J-HEALTH^25^** | |  | **At-HOME^26^** | |  | **HONEST^27^** | |
| --- | --- | --- | --- | --- | --- | --- | --- | --- | --- |
| **Type of study** | | PMS, Losartan | |  | PMS, Azelnidipine | |  | PMS, Ormesaltan | |
| **Year for enrollment** | | 2000 | |  | 2006-2007 | |  | Registered Oct 2009 to Sep 2010 | |
| **Subject settings** | | 1,011 medical institutions | |  | 1,011 medical institutions | |  | clinics and hospitals | |
| **n** | | 4596 (patients with HBP) | |  | 4,852 | |  | 21,341 | |
| **Inclusion criteria** | | Patients who are not under treatment with any antihypertensive 1 month before Losartan is prescribed | |  | Outpatient with hypertension, no previous usage of study drug Home SBP > 135 mmHg and  office SBP > 140 | |  | -Olmesartan-naive outpatients with essential hypertension  -No restrictions were placed on prior antihypertensive drug treatment, with the exception of prior use of combination antihypertensives during the study | |
| **Exclusion criteria** | | Patients who had previously been treated with Losartan, had stroke or myocardial infarction within the previous month | |  |  | |  |  | |
| **Age** | | 60.8 ± 11.7 | |  | 64.8±11.9 | |  | 64.9±11.9 | |
| **Male** | | 43.2% | |  | 47.1% | |  | 49.5% | |
| **Medical History** | **Cerebrovascular disease** | 0.0% | |  | 7.4% | |  | 6.6% | |
|  | **Ischemic heart disease** | 0.0% | |  | 11.3% | |  | 4.5% | |
|  | **Diabetes mellitus** | Unspecified | |  | 17.8% | |  | 20.5% | |
|  | **Renal disease** | Unspecified | |  | 4.1% | |  | 20.1% | |
|  | **Dyslipidemia** | Unspecified | |  | 33.8% | |  | 44.6% | |
| **Timing** | | Baseline | After (6 months) |  | Baseline | After (16 week) |  | Baseline | After (16 week) |
| **Proportion of patients with at least one antihypertensives** | | 100.0% | Unspecified |  | 54.6% | Unspecified |  | 50.4% | Unspecified |
| **Number of antihypertensives** | | Unspecified | Unspecified |  | Unspecified | Unspecified |  | 1.57 ± 0.7 | 1.6 ± 0.8 |
| **Class of antihypertensives** | **CCBs** | Unspecified | - |  | 100.0% | - |  | 33.9% | 39.3% |
|  | **ACEi** | Unspecified | - |  | 5.4% | - |  | 1.4% | 1.4% |
|  | **ARB** | 100.0% | - |  | 35.9% | - |  | 100.0% | 97.6% |
|  | **Diuretics** | 0.0% | - |  | 5.6% | - |  | 4.5% | 6.6% |
|  | **Alpha blocker** | 0.0% | - |  | 3.2% | - |  | 2.0% | 2.4% |
|  | **Beta blocker** | 0.0% | - |  | 6.9% | - |  | 6.0% | 6.5% |
| **Blood pressure**  **(mean±SD mmHg)** | **Morning home SBP** | 160.1 ± 17.8 | 138.7 ± 10.6 |  | 156.9 ± 16.4 | 137.7 ± 13.3 |  | 151.6 ± 16.4 | 135.0 ± 13.7 |
|  | **Morning home DBP** | 93.1 ± 11.9 | 81.4 ± 8.1 |  | 89.7 ± 12.0 | 79.4 ± 9.7 |  | 87.1 ± 11.8 | 78.8 ± 9.9 |
|  | **Office SBP** | 166.9 ± 16.4 | 140.5 ± 9.6 |  | 157.5 ± 18.7 | 138.9 ± 15.5 |  | 153.6 ± 19.0 | 135.5 ± 15.3 |
|  | **Office DBP** | 95.7 ± 11.0 | 81.4 ± 7.4 |  | 89.1 ± 13.3 | 78.9 ± 10.8 |  | 87.1±13.4 | 77.5 ± 10.9 |
| **Proportion of uncontrolled BP** | **Morning home BP**  **(>135 or >85 mmHg)** | 93.5% | 63.6% |  | 93.4% | 56.7% |  | 86.5% | 49.2% |
|  | **Office BP**  **(>140 or >90 mmHg)** | 98.1% | 56.7% |  | 87.1% | 43.9% |  | 80.3% | 36.9% |
| **BP phenotype*** | **Well-controlled** | 0.6% | 23.8% |  | 3.5% | 32.2% |  | 7.9% | 38.9% |
|  | **White-coat hypertension** | 5.9% | 12.6% |  | 3.1% | 11.1% |  | 5.6% | 11.9% |
|  | **Masked hypertension** | 1.3% | 19.5% |  | 9.4% | 23.9% |  | 11.8% | 24.2% |
|  | **Sustained hypertension** | 92.2% | 44.1% |  | 83.9% | 32.8% |  | 74.7% | 25.0% |

*CCBs: calcium channel blockers; ACEi: angiotensin converting enzyme inhibitors; ARB: Angiotensin II receptor blockers; SBP: Systolic blood pressure; HT: Hypertension; BP: Blood pressure.*

**BP phenotype Well-controlled: (home blood pressure < 135/85 mmHg and office blood pressure < 140/90 mmHg); White coat hypertension (home blood pressure < 135/85 mmHg and office blood pressure > 140/90 mmHg); Masked hypertension (home blood pressure > 135/85 mmHg and office blood pressure < 140/90 mmHg); Sustained hypertension (home blood pressure > 135/85 mmHg and office blood pressure > 140/90 mmHg).*

**Supplementary 3. Distribution of hypertensive patients based on combination of home and office systolic blood pressure level in Japan**

|  | | **Home SBP [mmHg]** | | | | |
| --- | --- | --- | --- | --- | --- | --- |
|  |  | **<124** | **125-134** | **135-144** | **145-154** | **>155** |
|  | **Office SBP [mmHg]** |  |  |  |  |  |
| **Resistant hypertension** | **>160** | 0.0% | 3.4% | 3.4% | 4.1% | 12.3% |
|  | **150-159** | 0.7% | 0.7% | 3.4% | 6.2% | 2.7% |
|  | **140-149** | 5.5% | 5.5% | 12.3% | 2.1% | 1.4% |
|  | **130-139** | 3.4% | 10.3% | 4.8% | 0.7% | 0.7% |
|  | **<129** | 6.2% | 6.2% | 1.4% | 1.4% | 1.4% |
| **3+ antihypertensives** | **>160** | 0.2% | 1.7% | 2.2% | 2.8% | 5.6% |
|  | **150-159** | 0.4% | 0.9% | 1.7% | 3.0% | 2.4% |
|  | **140-149** | 1.9% | 3.9% | 6.7% | 2.6% | 0.9% |
|  | **130-139** | 6.7% | 12.3% | 7.6% | 4.3% | 1.1% |
|  | **<129** | 12.1% | 12.3% | 3.2% | 2.8% | 0.7% |
| **2 antihypertensives** | **>160** | 0.2% | 0.5% | 0.7% | 0.8% | 2.9% |
|  | **150-159** | 0.2% | 0.8% | 2.1% | 2.4% | 1.2% |
|  | **140-149** | 1.6% | 4.5% | 6.6% | 3.4% | 1.3% |
|  | **130-139** | 6.3% | 15.9% | 11.4% | 3.6% | 1.8% |
|  | **<129** | 13.5% | 11.2% | 5.2% | 1.4% | 0.7% |
| **1 antihypertensives** | **>160** | 0.2% | 0.7% | 0.8% | 0.8% | 2.3% |
|  | **150-159** | 0.5% | 1.4% | 2.4% | 2.3% | 1.5% |
|  | **140-149** | 1.8% | 4.3% | 7.3% | 3.1% | 0.9% |
|  | **130-139** | 6.9% | 17.1% | 12.2% | 3.1% | 1.4% |
|  | **<129** | 11.5% | 11.9% | 3.8% | 1.5% | 0.5% |
| **No antihypertensives** | **>160** | 0.2% | 0.7% | 1.1% | 1.3% | 3.2% |
|  | **150-159** | 0.5% | 1.4% | 2.2% | 3.6% | 1.7% |
|  | **140-149** | 1.3% | 3.1% | 9.0% | 3.8% | 1.3% |
|  | **130-139** | 5.1% | 16.2% | 11.7% | 3.6% | 1.9% |
|  | **<129** | 11.2% | 10.9% | 3.3% | 1.4% | 0.5% |
| **Total** | **>160** | 0.2% | 0.7% | 0.9% | 1.0% | 2.8% |
|  | **150-159** | 0.5% | 1.3% | 2.2% | 2.7% | 1.5% |
|  | **140-149** | 1.6% | 4.0% | 7.6% | 3.3% | 1.1% |
|  | **130-139** | 6.3% | 16.4% | 11.7% | 3.4% | 1.6% |
|  | **<129** | 11.8% | 11.5% | 3.9% | 1.5% | 0.5% |

*SBP: Systolic blood pressure.*

**Resistant hypertension is defined as having an office blood pressure of 140/90 mmHg or more while taking three antihypertensive medications, including diuretics, or taking four or more antihypertensive medications regardless of blood pressure level.*
